# Workaholism Scales: Some Challenges Ahead

**DOI:** 10.3390/bs13070529

**Published:** 2023-06-23

**Authors:** Leandro Gonçalves, Jéssica Meneses, Simão Sil, Tatiana Silva, António C. Moreira

**Affiliations:** 1 DEGEIT—Department of Economics, Management, Industrial Engineering and Tourism, University of Aveiro, Campus Universitário de Santiago, 3810-193 Aveiro, Portugal; leandro.goncalves@ua.pt (L.G.); jessicameneses@ua.pt (J.M.); simaopedrosil@ua.pt (S.S.); tatiana.silv@ua.pt (T.S.); 2 INESCTEC—Institute for Systems and Computer Engineering, Technology and Science, Campus da Faculdade de Engenharia da Universidade do Porto, Rua Dr. Roberto Frias, 4200-465 Porto, Portugal; 3 NECE-UBI—Research Center for Business Sciences, Universidade da Beira Interior, 6200-209 Covilhã, Portugal; 4 GOVCOPP—Research Unit on Governance, Competitiveness and Public Policies, University of Aveiro, 3810-193 Aveiro, Portugal

**Keywords:** workaholism, work addiction, scales, the systematic literature review

## Abstract

Although extensively used in the academic literature, workaholism as a concept has been explained in different ways, which has influenced the development and use of some measurement tools. As such, this article aims to address the subject through a systematic study review focusing on articles where the main objective was to develop, adapt, or analyze the psychometric properties of a workaholism scale. The main purpose is to describe the state of the art concerning workaholism measurement tools, highlighting trends and research perspectives for further research. In essence, this study may serve as a summary and starting point for scholars interested in measuring workaholism. It was observed that the discrepancy concerning the definition of workaholism has resulted in scales that attempt to evaluate diverging conceptualizations. Moreover, each scale has been readapted when tested in different countries. For further investigations, it is important to converge the concept of workaholism and validate the scales across differing contexts, regarding the industry, culture, and country of the sample.

## 1. Introduction

Oates [1] was among the first in coining the term ‘workaholism’ in the academic literature, which was first described as an “addiction to work, the compulsion or uncontrollable need to work incessantly” [1,2]. Andreassen, Griffiths, et al. [3] and Salvador et al. [4] developed their own definition of workaholism, characterizing it as an addiction to work that involves negative outcomes at the individual, interpersonal, and organizational levels, significantly affecting the life of a worker. Consequently, individuals who exhibit high levels of workaholism tend to prioritize work over leisure activities, neglect their relationships with others, and disregard their own health [4]. This definition implies that workaholism is a subtype of excessive work engagement, which involves two different dimensions [2]: time and effort. Most definitions interpret workaholism as a chronic pattern of overindulgence in working for long hours, being engaged and self-observed in work more than is demanded by implicit and explicit norms [2].

Workaholics are described as being highly involved in work, feeling compelled to work by inherent pressures and experiencing a low enjoyment when working [5]. Consequently, they struggle to disengage from work due to their internal drive to work intensely hard [6].

Work addiction has been described as a dysfunctional approach to work [7], which can be understood as an excessive and compulsive urge to work [8]. The earliest studies on work addiction date back to the early 1990s, concerning triggers for health, family, and emotional problems [7]. Since then, there has been a growing interest in work addiction due to its impacts in social functioning, interpersonal relationships, and personal wellbeing [7]. However, there is a lack of solid guidelines for preventing and recovering from work addiction [7]. However, the terms ‘work addiction’ and ‘workaholism’ have been used interchangeably, with work addiction being more prevalent on the core addiction literature, while the workaholism is related to working excessively, regardless their positive or negative outcomes [9].

Two features stand out in the original concept of workaholism: working excessively and working compulsively [10]. Working excessively pertains to a behavioral tendency reflected in an extraordinary amount of time that is devoted to work activities [10]. There are multiple reasons why individuals strongly dedicate their time to work, but in some cases, workers are influenced by a compulsive inner drive to work excessively [6]. Thus, working excessively encompasses the behavioral dimension of workaholism [6], whereas working compulsively refers to the cognitive or mental dimension of workaholism, which translates in an obsession to work excessively hard [10].

Notably, workaholism is regarded as being a ‘bad type’ of working hard, whereas work engagement has the reputation of being a ‘good type’ of working hard [3]. Hence, work engagement is associated with a positive work-related behavior, where workers devote their high energy to carry out tasks that result in high levels of dedication and focus on their work [4]. Therefore, individuals who possess more energy and enthusiasm in carrying out work activities exhibit high levels of work engagement [4]. This results in better employee performance and enhanced wellbeing within organizations [4]. Although engaged workers are highly involved with work, for them, work is enjoyable [3]. In contrast, workaholics, despite being heavily invested in work, are driven by a compulsive motive that is characteristic as an addiction [3]. However, it is implied in several studies that excessive work engagement can cause negative side-effects and even turn engaged employees into workaholics, as they can be so immersed in their work that they can have difficulties in maintaining personal relationships and forget to rest [11]. Due to the overlapping features between work engagement and workaholism, they are often not clearly distinguished by researchers, which should be more adequately handled [12].

Although research on workaholism is not new, various scales have been used to measure it. However, some of them not only focus more on work addiction than workaholism, but also overlap with work engagement, working excessively, or working compulsively, without distinguishing these different constructs [3,10]. Moreover, there are several concepts that are important for work engagement, but they can overlap with workaholism, but are totally different, supporting the confusion surrounding workaholism [11,12,13,14]. Furthermore, some of those scales use multi-factor dimensions, whereas others use a single-factor dimension. As such, this article aims to review the literature seeking to uncover the main workaholism scales used to measure workaholism and compare them to analyze their main key characteristics. With this review, we seek to contribute to uncover those different workaholism scales in order to understand the different dimensionalities. By no means, we seek to homogenize or come up with a single, unified tool.

Following this introduction, the article is structured in five sections. Section 2 describes the methodology implemented. Section 3 characterizes the articles selected in the previous section and analyzes the main scales found. Section 4 presents a discussion of the results. Finally, Section 5 presents the main conclusions derived from the analysis.

## 2. Methodology

This paper follows the guidelines of the Systematic Literature Review (SLR), based on instrumental articles and reviews advanced to further consolidate, develop, and validate scales for measuring workaholism. The SLR conducted in this article follows the PRISMA protocol [15], typically employed in healthcare research. As the number of stages of the PRISMA protocol varies according to the authors [16,17], the following stages were put through to conduct the analysis [3,18]:

- First, a review was conducted based on the research objectives.
- Secondly, manuscripts were selected based on strict criteria to guide the search in the Scopus database and to identify the scales used to measure workaholism.
- Finally, relevant information extracted from the selected articles was analyzed, and the results were discussed.

**Figure 1:**
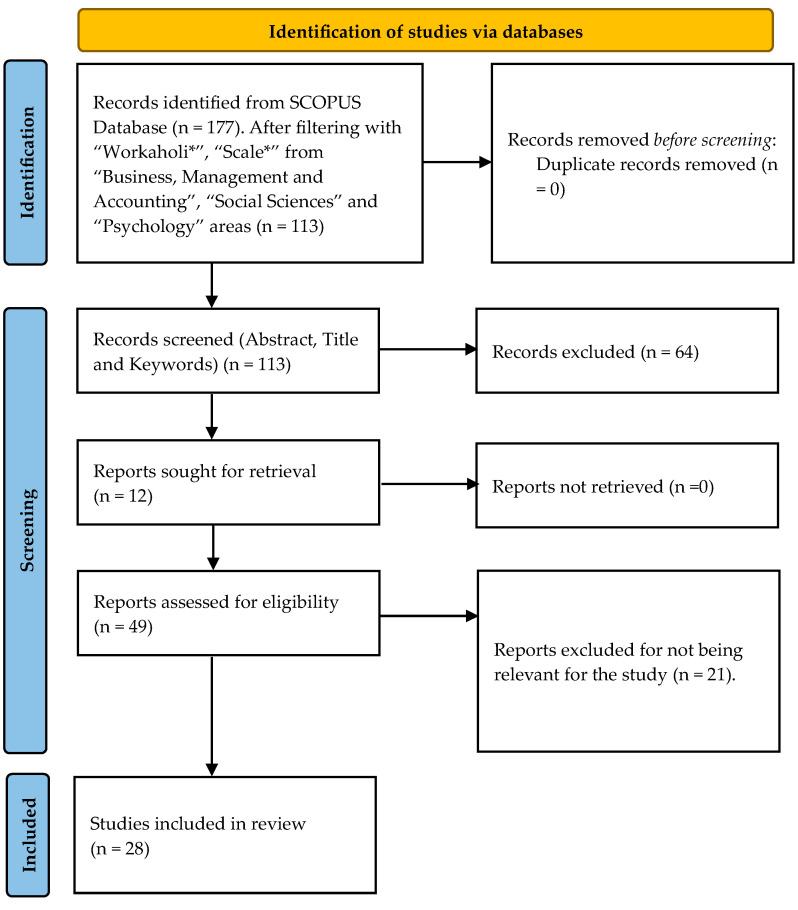
PRISMA flow diagram.

First and foremost, the SCOPUS database was chosen to search for scientific articles, and reviewed them as reliable, curated databases, covering peer-reviewed articles from quality-assessed journals [19,20]. The search keywords used in this review were “Workaholic*” and “Scale*,” aiming to capture different words related to “workaholic” and “scales”. With this search, the aim was to be as inclusive as possible. Given that workaholism is a psychological aspect, both the “Business, Management and Accounting”, “Social Sciences”, and “Psychology” areas were chosen under the “Subject area” option to restrict the analysis. The criteria sought to search for manuscripts pertained to the focus of the analysis: behavioral aspects with consequences for managers. Moreover, the document types were restricted to “Article” and “Review,” from journal articles, as those are considered as yielding the highest impact in terms of credibility and the most validated knowledge [21]. There were no restrictions regarding the publication year. Similar approaches were also used before [22,23]. The result of the whole process is shown in Figure 1.

This search strategy yielded 113 articles. The articles underwent a series of filters, with the objective of finding the best suited to the current review. As previously mentioned, only studies that have the objective of developing, analyzing, and validating the workaholism scales were considered suitable. As such, if the core of the manuscript was not workaholism, it was excluded. Studies that, for example, use the scales to just use the construct of workaholism as a variable in a determined sample/model were excluded.

In a first step, the title, keywords, and abstracts of each article/review were analyzed, resulting in three categories: accepted (articles that were used in the review); excluded (articles that are not anymore under consideration); and unconfirmed (articles that need a more thorough examination for content suitability). After this step, 37 articles were accepted, 12 unconfirmed, and the rest (64) were excluded.

The second step consisted of analyzing and discussing the inclusion of the unconfirmed manuscripts, leading to six being confirmed and six being excluded. For that, all authors read the manuscripts and the final decision (inclusion/exclusion) was taken on a consensual basis. In a final step, all accepted manuscripts were fully scrutinized, 15 of them being excluded for not analyzing workaholism or for the content being outside the scope of this study. Similar approaches were also used before [22,23]. Therefore, the outcome is a final sample of 28 suitable articles to be analyzed under this SLR.

## 3. Results

### 3.1. General Characterization of the Sample

The 28 articles included in this analysis were published in 25 different outlets, indicating that this topic is widely analyzed across different sources. Only three journals published two articles each, while the remaining journals published one article each. These data can be observed in Table 1.

As shown in Table 2, while the first article included dates from 1992, there is a clear rise in academic attention over time, since more than 30% (32.14%) of the articles relating to workaholism scales have been published from 2020 onwards. Moreover, the total global citations (TGCs) score was assessed to inspect the relevance of the articles. TGC reflects the number of times an article has been cited in the SCOPUS database, while total local citations (TLCs) denote the number of citations by other articles/authors within the selected literature [24]. Clearly, as shown in Table 3, there are six articles with more than 100 citations, while only six articles have a TLC score higher than 6. This suggests that these six articles are the most known within the workaholism literature. Of those six articles, Spence and Robbins [25] stand out, with a TLC score of 12.

### 3.2. Analysis of the Main Scales

This section describes, in detail, the various scales used to measure workaholism according to the SLR carried out, namely, the Dutch Work Addiction Scale (DUWAS), WorkBAT, Work Addiction Risk Test (WART), Bergen Work Addiction Scale (BWAS), and Multidimensional Workaholism Scale (MWS). The presentation of these scales is in chronological order, according to their date of creation.

#### 3.2.1. Work Addiction Risk Test (WART)

The first tool for measuring workaholism, known as the Work Addiction Risk Test (WART), was developed in 1989 by Robinson [44]. Initially, Robinson advocated for a single factor solution by combining 25 items into a comprehensive score. Later, Robinson and Post [45] proposed a five-factor solution, which includes Overdoing, Self-Worth, Control-Perfectionism, Intimacy, and Future Reference/Mental Preoccupation. These five factors are considered the major symptoms of work addiction [2,45].

Robinson [44] further refined the WART Scale and categorized workers as addicts, pseudo-addicts, and non-addicts. Flowers and Robinson [46] supported the five-factor solution, which consists of Compulsive Tendencies (nine items), Control (seven items), Impaired Communication/Self-Absorption (five items), Inability to Delegate (one item), and Self-Worth (two items). These five dimensions form of the factor structure of the WART Scale. The validity and reliability of the five-factor solution proposed by Flowers and Robinson [46] were confirmed and validated by Taris et al. [27] using a Dutch sample. The main findings reveal that the 25-item Dutch version of the WART is a robust instrument for measuring work addiction. The Compulsive Tendencies dimension was found to be a strong indicator of workaholism, as the study demonstrates that the association between the nine-item Compulsive Tendencies dimension and the 25-item WART scale, was high and, therefore, it appeared that both retained very similar relationships with other concepts. All things considered, it is advised that, by only focusing on the compulsive tendencies dimension, little information is lost. Thereby, the results show that it is not necessary to make use of the full WART to measure workaholism, as the compulsive tendencies dimension already provides a good indication to identify the degree to which participants experience workaholism [27].

Authors from different countries have proposed different factor structures. Clark et al. [31], using a convenience sample of American students who worked 36 h per week, identified only three factors: Impatience, Compulsion to Work, and Polychronic Control. Andreassen, Hetland, et al. [2], aiming to replace the student convenience sample with Norwegian employees, failed to replicate the five-factor solution. Instead, they found support for a four-factor solution. The first factor, named Overwork, encompasses overwork-related items and shows a broad overlap with the Overdoing factor identified by Robinson and Post [45]. The second factor, Control-Perfection, includes items related to perfectionism and distractibility, aligning with the Control-Perfectionism scale by Robinson and Post [45]. Factor three, Intimacy/Work-Family Interface, mirrors the two items of the Intimacy subscale by Robinson and Post [45]. The fourth factor is related to Impatience, which also covers the Self-Worth subscale by Flowers and Robinson [46]. In this way, Andreassen, Hetland, et al. [2] argue that the factor structure of the WART scale remains unclear, with previous studies mentioning it as well.

Urbán et al. [9] revised the factor structure of the WART, emphasizing the lack of consensus in previous studies. They suggested that differences in factor structures may stem from variations in cultural contexts and sample characteristics. To develop a more robust factor structure, referred to as WART-R, Urbán et al. [9] tested one, four, and five-factor models of the WART scale. None of them presented a satisfactory fit with a sample of individuals working at least 40 h a week. The best-fitting model identified four factors: Overcommitment, Impatience, Hardworking, and Salience, containing 17 items. Overcommitment corresponds to the compulsive tendency, the first factor in the original five-factor model. Impatience matches the control factor in the original factor solution. In the 5-factor solution, the first, third, fourth, and fifth factors were reconceptualized as Hardworking and Salience in the revised WART-R model [9].

#### 3.2.2. Workaholism Battery (WorkBAT)

The Workaholism Battery (WorkBAT), initially developed by Spence and Robbins [20], employs a rational–deductive methodology to generate a three-dimensional model of workaholism [28,30], involving work involvement (WI), driveness (D), and work enjoyment (E). The original scale of WorkBAT comprises a 25 item-scale that also takes a trait approach, answered on a 5-point Likert scale [3,33]: driveness, a seven-item dimension, assesses a person’s inner pressure to work and a feeling of being compelled to work; work enjoyment, a 10-item dimension, measures the extent to which individuals enjoy their work; and work involvement, an eight-item dimension, measures one’s need to be productive and efficient both at and off work [33].

A disadvantage when using WorkBAT Is that workaholism cannot be clearly differentiated from work engagement. This is because workaholism encompasses positive emotions, which means that the concept of workaholism measured by WorkBAT shares with work engagement both behavioral and emotional aspects, and these two concepts must be differentiated between each other because negative feelings, such as anxiety, anger, and disappointment, are closely related to workaholism, whereas positive feelings, such as joviality and self-confidence, relate to work engagement [42]. Another limitation is that WorkBAT is primarily theoretical and does not incorporate the core components of addiction, such as tolerance, withdrawal, conflict, salience, mood modification, and relapse [9].

Studies on the factorial validity of the WorkBAT have failed to confirm Spence and Robbins’ [25] three-factor model of workaholism [29,30,39]. As a result, several studies proposed alternative two-factor structures of the WorkBAT, consisting of a revised D scale and a revised E scale, while eliminating the WI dimension [29,33]. McMillan et al. [28] not only reported the absence of the three-factor structure of the WorkBAT, but also found a low level of internal consistency [42]. Other studies have also highlighted low reliability in the WI dimension and the low relevance of the E dimension to the concept of workaholism has been questioned [42]. In sum, the construct validity of the WorkBAT has not yet been convincingly established, particularly given the limited sample sizes and the clustering process used in the construction to the original scale [28].

Consistent with the literature, the WorkBAT scale has been adapted in three languages: Spanish [36], traditional Chinese [33], and Japanese [5]. Empirical data in these studies did not support the three-factor model proposed by Spence and Robbins [25]. However, the WorkBAT scale captures both positive and negative aspects and has acceptable psychometric properties of workaholism, thus providing a comprehensive picture of workaholism. The variations found in these studies was that the WorkBAT scale was reduced from 25 items to 19 items in Boada-Grau et al. [36], utilizing two factors (D and E) with 12 and 7 items, respectively. Huang et al. [33], while employing the traditional 25 items, applied a 7-point Likert-type scale instead of a 5-point Likert-type scale originally used by Spence and Robbins [25]. Lastly, Kanai et al. [5] added 39 work-related behavioral items—to assess the subjects’ level of involvement, at work, work stress, time commitment, perfectionism, and non-delegation—to the original 25 items using a 4-point Likert-type scale.

#### 3.2.3. Dutch Work Addiction Scale (DUWAS)

Work addiction is understood as an excessive and compulsive urge to work [8]; however, studies using the DUWAS scale often use the words ‘workaholism’ and ‘work addiction’ interchangeably. To deepen the study of the subject of work addiction, Schaufeli, as wel as Taris et al. [47], developed the DUWAS scale. The items of this scale are based on the WART and WorkBAT scales [9], as well as the original conceptualization of workaholism developed by Oates [1,10], which can be described as “the uncontrollable need to work excessively hard” [8]. Concerning the analysis of the subject, the DUWAS scale distinguishes working excessively (WE) and working compulsively (WC), where working excessively uses items from the Compulsive Tendencies Scale of the WART and working compulsively adopts items from the Drive Scale of the WorkBAT [10]. This scale has the goal of evaluating work addiction among workers [40]. The DUWAS scale can be characterized as “a brief two-dimensional self-report instrument that includes two correlated subscales of five items each: working excessively (WE) and working compulsively (WC)” [8,26]. Workaholism can be assessed using two different versions of the DUWAS scale [10]: the original survey with seventeen items and the final version with ten items, five for each of the two dimensions. This last interpretation of the scale is often called DUWAS-10 [6] and aims to improve the scale’s psychometric properties while reducing its length [10]. Furthermore, after conducting a study in the Brazilian context, Salvador et al. [4] claim that a unifactorial structure for the scale is optimal, providing good psychometric properties, internal consistency, and reliability.

The DUWAS scale has been adapted and combined with other scales to validate and analyze workaholism in specific international contexts. For instance, Vazquez et al. [40] used the DUWAS-16 version of this scale to investigate workaholism in Brazil. Mir et al. [38] translated the DUWAS-10 version to assess its validity in Pakistan. Huyghebaert-Zouaghi et al. [37] employed this scale alongside the Utrecht Work Engagement (UWES) scale in order to explore the relationship between work engagement and workaholism. 

When analyzing the convergence between self and observer ratings of workaholism, Falco et al. [34] developed two additional versions of this scale: the DUWAS-OR (observer rating) and DUWAS-R (self-report). The earlier Italian adaptation of the original DUWAS (17 items) is described in Kravina et al. [48]. Additionally, Sharma and Sharma [39] used a three-construct version of the DUWAS scale (‘working compulsively’ with seven items, ‘working excessively’ with nine items and ‘overwork’ with four items).

This DUWAS scale has been translated into various languages, resulting in versions tailored to specific countries, such as the Italian version [35]. The Working Excessively (WE) dimension pertains to the tendency to dedicate an exceptional amount of time to work-related activities. Working Compulsively (WC) refers to the cognitive aspect of work addiction, characterized as an obsession with work [7]. Therefore, WE is closely related to the behavioral aspects of workaholism, whereas WC is linked to cognition [6].

Studies conducted in several countries with the DUWAS scale have consistently supported its validity as a two-factor structure, demonstrating good psychometric characteristics and suitability for analyzing of work addiction [10,40]. Furthermore, it has been shown that the DUWAS scale has high test-retest reliability [10].

Using the DUWAS scale, Schaufeli, Wijhe, et al. [8] concluded that workaholics and compulsive workers have similar personality traits and outcomes and that workaholics often have more energy than compulsive workers, who similarly have the urge to work while lacking the energy. Besides, workaholics are often more prone to burnout than compulsive workers [8]. De Beer et al. [6] confirm that workaholism is negatively associated with work engagement, performance, and organizational commitment, while positively related to mental distress and burnout. Littman-Ovadia et al. [10] conclude that workaholism is better understood as a personal disposition rather than a situational response. Balducci et al. [32] claim that workaholism, particularly the WE dimension, is positively and strongly associated with job demands and is linked to psychological and physical reactions.

#### 3.2.4. Bergen Work Addiction Scale (BWAS)

As previously mentioned, the lack of consensus in the definition of workaholism blurs the line between this term and work addiction. Thus, it is natural for some authors to view workaholism as a behavioral addiction [43].

Taking an addiction perspective on workaholism, Andreassen, Griffiths, et al. [3] analyzed multiple existent workaholism scales, namely, DUWAS, WART, and WorkBAT. They found that these scales, in general, have not been strongly grounded in addiction theory, leading to a lack of face validity [3]. Dissatisfied with this predicament, a new shorter scale was developed, with the objective of being mostly based on the addiction perspective to facilitate its usage in clinical contexts. This scale is named the Bergen Work Addiction Scale (BWAS) and can be classified as a one-dimensional scale (being the first one-dimensional workaholism scale), comprising seven items, each representing the core elements of addiction, being Cognitive salience, Tolerance, Mood modification, Relapse, Withdrawal, Conflict, and Health problems caused by the excessive engagement in the activity [3]. Additionally, the scale also addresses three dimensions of workaholism as defined by Ng et al. [49], which include affection, cognition, and behavior. The BWAS scale tackles these dimensions by evaluating the possibility that workaholics may use work to escape personal problems, long working hours, and their lasting obsession with working. Unlike other scales, while being based on these sub-elements, the BWAS is unidimensional, only presenting workaholism as its dimension.

The WART, WorkBAT, and DUWAS-10 scales have been used to investigate the convergent and discriminant validity of BWAS. Initially, Andreassen, Griffiths, et al. [3] found weak correlations between BWAS and specific subscales of the WART and WorkBAT that pertain to the positive aspects of workaholism. In contrast, stronger correlations were observed with subscales related to negative aspects, which were expected, given that the BWAS is based on the principles of addiction. Molino et al. [43] advanced the validation process by testing the correlation between BWAS and DUWAS-10, which is the more commonly used workaholism scale. Their study had a two-fold objective: to validate the psychometric properties of BWAS while also validating an Italian version [43]. The use of DUWAS and its already developed Italian version was thus imperative to reach this objective. After testing convergent validity, it was determined that BWAS and DUWAS, both the English and Italian versions, are two measures of the same construct. Additionally, the discriminant validity of the BWAS was also confirmed through the usage of a Work Engagement (WE) scale. DUWAS showed a positive correlation with WE, while BWAS had a negative correlation, as expected, since DUWAS captures engagement and BWAS does not [43].

Having good psychometric properties, as confirmed by Molino et al. [43], the BWAS is a validated scale for measuring workaholism. Its primary strength is its brevity, making it highly practical for clinical assessments. Its convergent and discriminant validity have been tested with other workaholism scales, which means that although it measures workaholism, it has sufficiently different properties to be considered an alternative. The suggested cut-off value proposed by Andreassen, Griffiths et al. [3] is also supported and validated by Molino et al. [43].

#### 3.2.5. Multidimensional Workaholism Scale (MWS)

Similar to the BWAS, the development of the Multidimensional Workaholism Scale (MWS) derives from the inconsistencies in the definition of workaholism. Clark et al. [31] argue that most of the mentioned workaholism scales suffer from construct contamination since, due to the absence of consensus, they include items that better reflect other constructs. Kim et al. [42] also state that the existing measures are constantly being altered, as attempts to replicate the factor structure developed in the original study ended up in failure. This poses significant challenges, as it creates obstacles in researching the impact and predictability of workaholism [31,42].

While the MWS shares the development vision of BWAS in relation to the current state of workaholism measures, Clark et al. [31] strictly separate workaholism from work addiction. They argue that, although the terms have been used interchangeably, they represent two different constructs and conditions, with work addiction being a more clinically oriented term.

With this in mind, the development of MWS not only proposes a new measure, but also a multidimensional definition of workaholism. Being the youngest fully developed scale to date, the MWS benefits from hindsight, as it is developed as an answer to decades of criticism and evaluation of the existing scales and addresses the raised issues and limitations. As Kim et al. [42] refers, while definitions of workaholism may vary, there are some attributes that are consistently mentioned. The MWS incorporates these attributes, encompassing a motivational dimension (inner pressure to work), cognitive dimension (uncontrollable thoughts about work), an emotional dimension (negative emotions when not working), and finally a behavioral dimension (excessive working) [26,29,31]. Aside from its original development study conducted in the US [31], the MWS has already been tested in two other different languages: Chinese [41] and Korean [42]. The validation of the scale in these three distinct languages and cultures strongly supports its applicability, as it demonstrates that, while different cultures have equally different definitions of what represent workaholism, the MWS clearly identifies workaholism in all of them. All the aforementioned studies have declared that the scale has very good psychometric properties, which is impressive, as it is a relatively recent measure.

The convergent and discriminant validities were also assessed. By using existing workaholism scales, being the DUWAS, WART, and WorkBAT, Clark et al. [31] successfully indicate that, including MWS, they are all measures of the same construct. Kim et al. [42] also state that the Korean version was expectedly related to the WorkBAT and DUWAS scales, while pointing out that Xu and Li’s [41] research suffered from a gap by not evaluating MWS against existing scales. While being related to these other measures, the MWS appears distinct from them, showing discriminant validity, as it confines all the core dimensions of workaholism, and it also has demonstrated incremental validity over them in the prediction of outcomes related to workaholism, such as emotional exhaustion [31,42]. Furthermore, all studies assessed that, while the MWS positively correlates with work engagement, they are uniquely related to the outcomes, which means that, even though they are measures of heavy work investment and, thus similar, they are very distinct measures [31,41,42].

The Multidimensional Workaholism Scale (MWS) is a 16-item tool that presents itself as a four-factor model, with each dimension comprising four items. Various studies have confirmed its optimal psychometric properties and robustness, though changes have been made to the original scale. For instance, Kim et al. [42] found that a 14-item version had better model fit, while Xu and Li [41] proposed a bifactor model. The differences observed could be due to cultural characteristics, as noted by Kim et al. [42]. While some researchers, such as Clark et al. [31] and Kim et al. [42], argue that the MWS is useful for evaluating the impact of each dimension on workaholism outcomes, Xu and Li [41] urge caution, particularly since the dimensions are uncorrelated in the bifactor model.

## 4. Discussion

This study review provided a substantial amount of information regarding workaholism scales, their theoretical backgrounds, and adaptations. Clearly, the concept of workaholism is multifaceted, with a lack of a clear, standardized definition.

The main evidence identified for each scale in the reviewed articles is introduced in an integrated framework in Table 4, while the original scales are listed in Table 5. As a result, other subtopics related to heavy work investment, such as work engagement, excessive working, compulsive working, and work addiction, often experience some overlapping, as their meanings can slightly vary depending on the sources. A good example of this phenomenon is the fact that some authors differentiate workaholism from work addiction, while others use both expressions interchangeably. The lack of a consensus between definitions leads to different interpretations of the concepts and, therefore, there have been a wide variety of approaches to measuring workaholism as a whole.

The WART scale, initially developed in 1989 by Robinson [44], consists of a single factor. However, he also proposed other scales that supported different five-factor solutions, both in 1994 [45] and 2002 [46]. In 2005, Taris et al. [27] attempted to validate the scale using a Dutch sample, finding that both the original 1989 version and the 2002 version were reliable measures of workaholism. The compulsive tendencies subscale was highlighted, as it was argued to be effective as the 25-item scale in measuring workaholism. Taris and Schaufeli would go on to develop the DUWAS scale. Based heavily on the notion of addiction, the WART scale is limited because it only captures the negative aspects of workaholism.

In 2019, the factor structure of the WART was reviewed [9], resulting in a more robust factor structure, renamed WART-R, because from all the previous factor solutions put forward, none of them showed a satisfactory fit in the sample of individuals working at least 40h per week. The best-fitting model identified four factors: overcommitment, impatience, hard work, and salience, encompassing a total of 17 items.

The WorkBAT scale was developed in 1992 by Spence and Robbins [25], containing a three-factor structure—work involvement (WI), driveness (D), and work enjoyment (E)—to measure workaholism. This scale differs from the WART scale, which aims to measure work addiction. This validity and reliability of the WorkBAT scale was tested in Japan in 1996, but the original structure was not supported, potentially due to sociocultural factors [5]. Kanai et al. [5] added 39 work-related behavioral items—to assess the subjects’ level of involvement, at work, work stress, time commitment, perfectionism, and non-delegation—to the original 25 items using a 4-point Likert-type scale.

In 2002, McMillan et al. [28] attempted to validate the WorkBAT scale in New Zealand, but internal inconsistencies were found, and the original structure was not supported. In 2010, Huang et al. [33] examined the factor structure of WorkBAT in a Chinese sample (using traditional Chinese) and failed to replicate the original structure proposed by Spence and Robbins [25], as well as the adapted versions developed by McMillan et al. [28] and Kanai et al. [5]. In their study, Huang et al. [33] used the traditional 25 items, but applied a 7-point Likert-type scale, instead of the original 5-point Likert-type scale. They also proposed a different version of the WorkBAT, consisting of a revised D scale, a revised E scale, and the elimination of the WI dimension.

In 2013, Boada-Grau [36] validated a Spanish version of the WorkBAT using a Spanish sample and found no support for the previous structure. They concluded that a scale reduced to two factors and 19 items performed better. In 2014, Andreassen, Hetland, et al. [2] conducted a study to investigate the three most commonly used scales—WorkBAT, WART, and DUWAS—in a cross-occupational Norwegian sample. While they found that the reliability of the tools was good and stable over time, the WorkBAT scale by Spence and Robbins [25] was not supported [2].

The DUWAS scale was developed by Schaufeli, Taris, et al. [33] and Schaufeli, Shimazu, et al. [26]. This scale, which builds upon the WorkBAT and WART scales [9], comprises two dimensions: working excessively and working compulsively. Although it has demonstrated good psychometric properties, the inclusion of the working excessively dimension has been criticized for not being associated with the fundamental aspects of addiction. 

In 2010, del Líbano et al. [29] analyzed the psychometric properties of the DUWAS scale in a Spanish and Dutch sample and confirmed its robustness and validity in both countries. In 2011, Schaufeli, Taris, et al. [47] and Schaufeli, Shimazu, et al. [26], the creators of the DUWAS, also validated it as a reliable tool to measure workaholism, using again a Dutch sample.

In 2012, Falco et al. [34] examined workaholism from the perspective of an observer and developed two versions of the DUWAS: the DUWAS-OR and DUWAS-R. The DUWAS-OR is a two-factor tool that demonstrated both convergent and discriminant validity. In 2013, Sharma and Sharma [39] explored the psychometric properties of the DUWAS scale in the Indian work environment and concluded that a three-factor solution, including an “overwork” dimension, was a valuable tool for the Indian context, particularly in the service setting. In 2014, Littman-Ovadia et al. [10] examined the psychometric properties of the Hebrew version of the DUWAS-10 and confirmed the validity of its two-factor structure, replicating the original findings in the Dutch data.

In 2016, the DUWAS-10 scale was translated to test its validity in Pakistan [38]. The DUWAS scale has been adapted to specific countries, including an Italian version [32]. In 2020, the DUWAS-16 version was used to study workaholism in Brazil, and the validity of the DUWAS scale as a two-factor structure was supported. Additionally, the WART scale was tested using only three factors of the scale (Impatience, Compulsion to Work, and Polychronic Control) [40]. In 2021, the DUWAS scale was adapted to specific countries, such as the Argentine version [7].

As recently as 2022, a study in the Brazilian context concluded that a single-factor structure for the DUWAS scale is optimal [4]. Furthermore, DUWAS-10 was validated and adapted for the South African financial services context [6]. In 2023, DUWAS-10 version was used together with the UWES scale to study the relationships between work engagement and workaholism [37].

Despite the focus in addiction exhibited by the WART and DUWAS, in 2012, Andreassen, Hetland, et al. [2] analyzed these scales, along with the WorkBAT, and realized that they were not developed with a strong enough basis in addiction. The DUWAS scale, in particular, was criticized for its “working excessively” dimension not being associated with the fundamental aspects of addiction. In response, the BWAS scale was created, utilising the component model of addiction, which provides a valuable framework for comprehending and identifying its features. The BWAS scale was also idealized to be more practical and easily used in clinical contexts, which required a shorter length. Therefore, it consists of a one-dimensional scale, containing seven items. Although it was found to not be sufficiently distinct from the Compulsive Tendencies subscale of the WART, this was expected, since the BWAS scale is strongly rooted in addiction theory, and most addictions have strong elements of drive and compulsion.

In 2022, Molino et al. [43] sought to validate the psychometric properties of the BWAS while validating the Italian version at the same time. The study found that the BWAS and the DUWAS, in both English and Italian versions, were measuring the same construct. However, when testing the discriminant validity of the BWAS and DUWAS through using a Work Commitment (Work Engagement) scale, the latter correlated positively and the former did not, which is expected, since the DUWAS captures commitment and the BWAS does not.

The most recent fully developed scale of workaholism is the MWS, which was developed in 2020 by Clark et al. [31] using samples of university students, blue-collar, and white-collar workers in the USA. The origin of this scale can be traced to the inconsistencies in the definition of workaholism. As can be observed in this section, with the exception of the WorkBAT, the scales are based on the notion of work addiction, and not workaholism itself. Clark et al. argue that there is construct contamination due to the inexistence of a consensus and sought to develop a scale that is distinct from work addiction and strongly based on workaholism. The original MWS scale was based on a four-factor model with 16 items [31], but it was adapted by Kim et al. [42] to a 14-item version due to the cultural characteristics of Korea. Although changes were made to the original scale, it is argued that the MWS scale has optimal psychometric properties and is robust. In addition, the usefulness of this scale to assess the impact of each dimension on the outcomes of alcoholism at work is advocated.

It is evident that there is no one-size-fits-all scale for measuring workaholism, and the choice of scale depends on the specific context. However, among the existing workaholism scales (e.g., WorkBAT and DUWAS), the MWS is closely related [42]. Unlike WorkBAT and DUWAS, the MWS scale demonstrates excellent psychometric properties. In fact, the MWS has been shown to have incremental validity over the DUWAS, WART, and WorkBAT scales in predicting emotional exhaustion, negative work-related rumination, and depressive symptoms [31]. Moreover, the MWS scale captures distinct features of workaholism compared to work engagement, which means that MWS has better construct validity vis-à-vis other measures of workaholism, because workaholism and work engagement are conceptually different, with workaholism being associated with negative emotions, such as anxiety, anger, and disappointment, while work engagement is linked to positive feelings, such as joviality and self-confidence [42]. This subjectivity surrounding the measurement of workaholism has also influenced the development scale constructs, as certain authors prioritize certain themes over others when creating items. Furthermore, the relevance of work engagement in the measurement of workaholism has not been consistent across scales, as some authors find it imperative to distinguish workaholism from work engagement due to the different emotional experiences associated with each construct. The inclusion of addiction theory-related items and psychometric considerations has been a common feature in these scales, even though the specific issues chosen to add into the constructs could be substantially different.

The strengths and limitations of sch scale analyzed is shown in Table 6. The number of dimensions, factors, and items analysed in each scale varies according to the authors. This is dependent on the author’s conceptualization of workaholism that each author has and follows—as some authors believe that workaholism is a multidimensional concept and others do not—and which dimension should workaholism be based on. Moreover, it depends on the purpose of the scale, for example, Andreassen, Griffiths, et al. [3], when creating BWAS, had the objective of developing a practical workaholism scale that could be used in a clinical context, which led them to propose a theory-driven cutoff for identifying individuals with work addiction. In contrast, other scales have employed data-driven cutoffs.

When comparing scales, it is clear that some are more commonly used than others, even though all of them undergo adaptations and translations, demonstrating their flexibility in various situations. These scales have been used to investigate workaholism in a wide variety of professional and demographic contexts, including both in public and private sectors and different hierarchical positions.

Another important aspect highlighted in this review is the role of emotions in workaholic behavior. Scales, such as WorkBAT and MWS, incorporate the assessment of emotions as a dimension to measure and better define workaholism. This enables the examination of workaholism in relation to negative emotions, such as anxiety and disappointment, while work engagement is associated with motivation and self-confidence.

The flexible nature of these scales not only allows their adaptation and use in a wide variety of environments and research purposes, but also often inspires the development of hybrid and new scales altogether. However, this can also be a challenge as the original forms of the scales are rarely re-validated and are frequently modified when used in new contexts, indicating potential cultural biases. Even though some authors noted this obstacle and tried to develop a solution, such as the creation of the MWS [31], it is clear that this scale has already suffered some adaptations.

- No consensus relating to the factor structure
- Its validity oscillates when used in different cultures.
- Focused on work addiction instead of workaholism, and thus only captures the negative aspects of workaholism

This constant change can be understood as both an obstacle and an opportunity. It highlights the dynamic nature of research on workaholism, which reflects the increasing complexity of work environments as a result of increasing external pressures and advancements in psychology and management-related research. Nevertheless, it would be beneficial to foster collaboration among researchers to establish more solid understandings on concepts concerning workaholism and develop strategies to efficiently prevent and mitigate negative addictive tendencies towards work.

## 5. Conclusions

Both TGC and TLC scores reveal that there are some scales have been heavily cited. However, the low TLC scores indicate limited cross-referencing among these scales, suggesting that some of those scales have evolved somehow independently and remain very fragmented. 

There is an evident discrepancy regarding the concept under analysis. As previously emphasized, there is a clear distinction between the concepts of work addiction and workaholism. However, some scales, such as WART, DUWAS, and BWAS, classify them as identical. This is because the authors disagree substantially on the explanation of the concept of workaholism, which resulted in the development of numerous divergent paths. The fragmentation of the research topic is also evident in the constructs developed for each scale. Although some constructs are based on different scales, each scale differs on what it is actually evaluating, with some being more related to Work Engagement, while others lean towards Work Addiction.

The results also indicate that some scales are context-dependent, which indicates that they need to be tested and validated in different contexts. Although some articles report on the industry analyzed, the lack of information regarding the industry in which the sample is analyzed is a disadvantage for the development of the scales. As is evident, different industries have distinct realities and exert different pressures on individuals. For example, the fast-paced pharmaceutical industry can differ significantly from the somehow slow-moving automotive industry. Therefore, it is imperative that comparisons are made between different industries at an international level.

Although efforts have been made to test and validate workaholism scales in different countries and cultures, every attempt has resulted in adaptations and modifications to the core scales. Even the most recent scale, the MWS, which had the objective of converging the workaholism concept, has already been modified due to this influence. Given that workaholism is perceived differently, it is important to develop a scale and compare it across various countries and cultures to identify different predispositions to workaholic behavior. This would provide a clearer understanding of the effects of workaholism. For example, countries with high levels of collectivism may differ substantially from countries with a high level of individualism. The same applies for countries with high or low masculinity scores compared to those with high femininity scores.

Those characteristics can be understood as obstacles or opportunities, highlighting the dynamic nature of workaholism research, which is subject to constant change. This could be attributed to increasingly complex working environments due to external pressures and advancements in psychology and management-related research. Nevertheless, it would be beneficial if more cooperation among researchers could more effectively solidify all concepts concerning workaholism and develop strategies that can efficiently prevent and mitigate negative addictive tendencies towards work.

Even though this review had specific goals and research parameters, it still has limitations concerning the study of workaholism scales. The use of a single database (Scopus) may potentially limit the number of articles and reviews on the subject. Future studies on workaholism scales could complement this study by including the Web of Science database. Future studies could include words related to workaholism, such as “work addiction”, since it is often used as a synonym. Another limitation is that this paper is based on research on core subject of “Business, Management and Accounting”, “Social Sciences”, and “Psychology” as the motivation was to understand workaholism among business managers. Another limitation is that the paper does not address the reliability and discriminant validity of the different constructs, as well as their psychometric properties that may influence the dimensionalities of the scales. 

It is clear that management should develop strategies that can be both effective at achieving goals and at promoting mental wellbeing of workers and managers alike. Assessing workaholism can contribute to organizational wellbeing, as preventing compulsive and excessive behavior towards work could lead to a better work environment and performance, influenced by job satisfaction, better working environment, and work–life balance.

To prevent addictive work tendencies, it is recommended to establish systems in which work–life balance is present. Furthermore, it is advised for organizations to focus more on quality and job performance instead of working hours, as well as in intrinsic motivation and drivers. This may underpin job satisfaction and reduce undesirable work-related emotions towards work such as high anxiety and feelings of pressure, ultimately reducing burnout in the long run.

## Figures and Tables

**Table 1:** Main Journals.

Journal	Number of Articles	% of Total
*Journal of Psychology: Interdisciplinary and Applied*	2	7%
*Journal of Career Assessment*	2	7%
*Current Psychology*	2	7%
Others	22	79%

**Table 2:** Evolution of articles over time.

Year of Publication	Number of Articles	Percentage of Total Articles	Cumulative Percentage
2020–2023	9	32.14%	32.14%
2016–2019	4	14.28%	46.43%
2012–2015	7	25.00%	71.43%
2009–2011	3	10.71%	82.14%
1992–2005	5	17.85%	100.00%

**Table 3:** Ranking of the articles based on TGC and TLC scores.

Author	Source Title	Quartile	TGC	TLC
Spence and Robbins [25]	*Journal of Personality Assessment*	Q1	593	12
Schaufeli, Shimazu et al. [26]	*Cross-Cultural Research*	Q1	310	8
Taris et al. [27]	*Applied Psychology*	Q1	200	6
Andreassen, Griffiths et al. [3]	*Scandinavian Journal of Psychology*	Q1	198	3
McMillan et al. [28]	*Journal of Occupational and Organizational Psychology*	Q1	141	6
Kanai et al. [5]	*Japanese Psychological Research*	Q2	121	6
Del Libano et al. [29]	*Psicothema*	Q1	84	6
Mudrack and Naughton [30]	*International Journal of Stress Management*	Q1	65	1
Andreassen, Hetland et al. [2]	*Journal of Managerial Psychology*	Q1	60	4
Clark et al. [31]	*Journal of Applied Psychology*	Q1	48	1
Balducci et al. [32]	*European Journal of Psychological Assessment*	Q2	40	3
Huang et al. [33]	*Journal of Psychology: Interdisciplinary and Applied*	Q1	26	2
Littman-Ovadia et al. [10]	*Journal of Psychology: Interdisciplinary and Applied*	Q1	26	3
Falco et al. [34]	*TPM—Testing, Psychometrics, Methodology in Applied Psychology*	Q2	21	0
Nonnis et al. [35]	*BPA Applied Psychology Bulletin*	Q4	15	0
Schaufeli, Wijhe et al. [8]	*Gedrag en Organisatie*	Q4	15	0
Boada-Grau et al. [36]	*Anales de Psicologia*	Q2	11	0
Urbán et al. [9]	*European Addiction Research*	Q1	11	0
Huyghebaert-Zouaghi et al. [37]	*Current Psychology*	Q2	8	0
Mir et al. [38]	*Pakistan Journal of Psychological Research*	Q4	7	0
Sharma and Sharma et al. [39]	*Global Business Review*	Q2	6	0
Vazquez et al. [40]	*Avaliacao Psicologica*	Q4	6	0
Xu and Li [41]	*Journal of Career Assessment*	Q1	5	1
Kim et al. [42]	*Journal of Career Assessment*	Q1	2	0
de Beer et al. [6]	*SAGE Open*	Q2	1	0
Molino et al. [43]	*Europe’s Journal of Psychology*	Q2	1	0
Omar et al. [7]	*Current Psychology*	Q2	1	0
Salvador et al. [4]	*Psicologia: Teoria e Pesquisa*	Q4	1	0

Note: The best quartile is based on the 2021 Citescore from the Scopus database.

**Table 4:** Main Findings of the scales.

Article	DUWAS	WorkBAT	WART	BWAS	MWS	Comparison between Scales
Andreassen, Griffiths et al. [3]		X		X		X
Andreassen, Hetland et al. [2]		X				X
Balducci et al. [32]	X					
Boada-Grau et al. [36]		X				
Clark et al. [31]					X	
de Beer et al. [6]	X					
del Líbano et al. [29]		X				
Falco et al. [34]	X					
Huang et al. [33]		X				X
Huyghebaert-Zouaghi et al. [37]	X					
Kanai et al. [5]		X				
Kim et al. [42]		X			X	X
Littman-Ovadia et al. [10]	X					
McMillan et al. [28]		X				
Mir et al. [38]	X					
Molino et al. [43]				X		X
Mudrack and Naughton [30]		X				
Nonnis et al. [35]	X					
Omar et al. [7]	X		X			
Salvador et al. [4]	X					
Schaufeli, Shimazu et al. [26]	X					
Schaufeli, Wijhe et al. [8]	X					
Sharma and Sharma [39]		X	X			
Spence and Robbins [25]		X				
Taris et al. [27]	X		X			
Urbán et al. [9]	X	X	X			X
Vazquez et al. [40]	X					
Xu and Li [41]					X	

**Table 5:** Original Scale Structure.

Authors	Country	Year	Name of Scale	Number of Factors	Title of Factors	Number of Items
Robinson [44]	USA	1989	Work Addiction Risk Test (WART)	5	1. Overdoing 2. Self-Worth 3. Control-Perfectionism 4. Intimacy 5. Future Reference/Mental Preoccupation	25
Spence and Robbins [25]	USA	1992	Workaholism Battery (WorkBAT)	3	1. Work Involvement (WI) 2. Driveness (D) 3. Work Enjoyment (E)	25
Schaufeli, Taris et al. [47]; Schaufeli, Shimazu, et al. [26]	Netherlands	2009	Dutch Work Addiction Scale (DUWAS)	2	1. Working Excessively (WE) 2. Working Compulsively (WC)	17
Andreassen, Griffiths et al. [3]	Norway	2012	Bergen Work Addiction Scale (BWAS)	1	1. Workaholism	7
Clark, Smith, and Haynes [25]	USA	2020	Multidimensional Workaholism Scale (MWS)	4	1. Motivational Dimension 2. Cognitive Dimension 3. Emotional Dimension 4. Behavioral Dimension	16

**Table 6:** Strengths and limitations for each scale.

Scale	Strengths	Limitations
Work Addiction Risk Test (WART)	WART is a solid instrument to measure work addiction.	No consensus relating to the factor structure Its validity oscillates when used in different cultures. Focused on work addiction instead of workaholism, and thus only captures the negative aspects of workaholism
Workaholism Battery (WorkBAT)	Captures both positive and negative aspects and has acceptable psychometric properties of workaholism, thus providing a comprehensive picture of workaholism. Focused on workaholism instead of work addiction, and thus captures both positive and negative aspects.	Workaholism cannot be clearly differentiated from work engagement. Largely theoretical and is not based on the core components of addiction. Low reliability of the WI dimension and the E dimension was criticized due to its low relevance to the concept of workaholism. Was modified several times, now comprising various distinct factor solutions Has not yet been convincingly established
Dutch Work Addiction Scale (DUWAS)	Good psychometric characteristics and adequacy for the analysis of work addiction High test-retest reliability Tested in multiple and very different contexts	Focused on work addiction instead of workaholism The DUWAS scale has been adapted and mixed several times. Not developed with a strong basis in dependency, which means that the scale lacks face validity when measuring work addiction
Bergen Work Addiction Scale (BWAS)	Good psychometric properties Short and thus very useful and practical in clinical examinations Has a validated cut-off value	Focused on work addiction instead of workaholism
Multidimensional Workaholism Scale (MWS)	Developed as an answer to decades of criticism and evaluation of the existing scales and addresses the issues and limitations raised Applicability of the scale in distinct cultures Excellent psychometric qualities Better construct validity than other measures of workaholism Focused on workaholism instead of work addiction	Still very new, lacks a significant amount of tests that the other scales have Although new, its factor structure has been altered.
